# Oxidative Stress and Carbonyl Lesions in Ulcerative Colitis and Associated Colorectal Cancer

**DOI:** 10.1155/2016/9875298

**Published:** 2015-12-28

**Authors:** Zhiqi Wang, Sai Li, Yu Cao, Xuefei Tian, Rong Zeng, Duan-Fang Liao, Deliang Cao

**Affiliations:** ^1^Department of Pharmacology, Division of Stem Cell Regulation and Application, State Key Laboratory of Chinese Medicine Powder and Medicine Innovation in Hunan (Incubation), and Key Laboratory of Colleges and Universities in Hunan Province for Cytobiology and Molecular Biotechnology, Hunan University of Chinese Medicine, Changsha, Hunan 410208, China; ^2^Department of Medical Microbiology, Immunology & Cell Biology, Simmons Cancer Institute, Southern Illinois University School of Medicine, 913 N. Rutledge Street, Springfield, IL 62794, USA

## Abstract

Oxidative stress has long been known as a pathogenic factor of ulcerative colitis (UC) and colitis-associated colorectal cancer (CAC), but the effects of secondary carbonyl lesions receive less emphasis. In inflammatory conditions, reactive oxygen species (ROS), such as superoxide anion free radical (O_2_^∙−^), hydrogen peroxide (H_2_O_2_), and hydroxyl radical (HO^∙^), are produced at high levels and accumulated to cause oxidative stress (OS). In oxidative status, accumulated ROS can cause protein dysfunction and DNA damage, leading to gene mutations and cell death. Accumulated ROS could also act as chemical messengers to activate signaling pathways, such as NF-*κ*B and p38 MAPK, to affect cell proliferation, differentiation, and apoptosis. More importantly, electrophilic carbonyl compounds produced by lipid peroxidation may function as secondary pathogenic factors, causing further protein and membrane lesions. This may in turn exaggerate oxidative stress, forming a vicious cycle. Electrophilic carbonyls could also cause DNA mutations and breaks, driving malignant progression of UC. The secondary lesions caused by carbonyl compounds may be exceptionally important in the case of host carbonyl defensive system deficit, such as aldo-keto reductase 1B10 deficiency. This review article updates the current understanding of oxidative stress and carbonyl lesions in the development and progression of UC and CAC.

## 1. Introduction

Reactive oxygen species (ROS) refer to a class of special oxygen chemical forms or oxygen-containing compounds that have much higher chemical activity than the oxygen. Oxidative stress (OS) occurs if the generation of ROS exceeds the defensive capability of the antioxidant system in the cell [1]. As a largest endocrine and immune organ, the intestinal tract abundant with microorganisms is important in stress response, such as oxidative stress [2]. Superoxide anion free radical (O_2_^∙−^) and nitric oxide free radical (NO^∙^) are two main endogenous reactive oxygen/nitrogen species (ROS/RNS), from which other reactive free radicals, such as hydrogen peroxide (H_2_O_2_), hydroxyl radical (HO^∙^), and peroxynitrite anion (ONOO^−^), are derived [3]. There are several sources of ROS in the digestive tract [4]. Luminal microbes produce a large amount of ROS; inside cells, superoxide anion, hydrogen peroxide, and hydroxyl radicals are produced as byproducts of mitochondrial respiration in aerobic metabolism and in cytochrome P450 detoxifying reactions; and in the process of chronic inflammation, a large amount of ROS is produced by neutrophil phagocytosis of bacteria, granular materials, or soluble irritants [5, 6].

In normal condition, intestinal ROS have bactericidal effects, participating in the intestinal defensive function. However, oxidative stress derived from excessive ROS production over the buffering capability of antioxidant defense in the host would cause lipid peroxidation, intestinal mucosal barrier damage, bacterial translocation, and inflammatory response [2, 7]. Ulcerative colitis (UC) is a type of chronic inflammatory bowel disease (IBD) in which oxidative stress plays a critical role in its pathogenesis and malignant progression to colorectal cancer (CRC) [8, 9]. UC affects the distal colon and rectum but often extends to the proximal colon and eventually to the whole colon. Clinically, patients with UC usually experience an intermittent course for a lifetime and colectomy is the only curative option [10, 11]. A worse scenario of UC is the increased risk of developing colorectal cancer, so-called colitis-associated colorectal cancer (CAC) [12]. This review article focuses on the oxidative stress and secondary carbonyl (lipid peroxide) lesions in the pathogenesis of UC and CAC.

## 2. Oxidative Stress and Carbonyl Lesions in Ulcerative Colitis

UC is essentially an immune-inflammatory disease. Inflammation is a process that consists of a series of protective responses, such as immune cell infiltration and cytokine expression, to eliminate pathogens/insults and initiate damage repair of the tissue. Acute inflammation is the immediate response of the body to pathogens and characterized with recruitment of leukocytes, particularly granulocytes. Chronic inflammation is a prolonged inflammatory process and characterized by simultaneous damage and healing of tissues at the inflammatory spot, resulting in a progressive shift of cell types. Therefore, chronic inflammation often leads to progressive diseases in the host [13].

Ulcerative colitis (UC) is a chronic inflammation described with remission and reactivation [10]. In active phase, UC is characterized with diffusive inflammatory cell infiltration and small intestinal mucosal crypt abscesses. In the inflammatory colon, mucosa, submucosa, and lamina propria are often infiltrated with neutrophils, lymphocytes, plasma cells, and eosinophils [14]. The infiltrated neutrophils produce a large amount of ROS, triggering oxidative stress, and proteolytic enzymes. The proteolytic enzymes and ROS act on endothelial cells and cause cell injury and subsequent epithelial barrier permeability and luminal pathogen invasion, which in turn exaggerate inflammatory cell infiltration and inflammatory damage, eventually leading to intestinal mucosal necrosis and ulceration [15]. Meanwhile, epithelial regeneration starts to cover the ulcerative area under stimulation of mitogenic cytokines and prostaglandins produced in inflammatory response. In this circumstance, intestinal mucosal hyperemia, edema, and hyperplasia polyps may appear.

Etiopathology of UC is complicated, including bacterial or viral infection, changes of colon microbiota, excessive immune response, and oxidative stress injury [16, 17]. Host genetic factors also play an etiological role in the development and progression of UC. It has been reported that the chromosomal loci 3, 7, and 12 in humans are associated with individual sensitivity to inflammatory bowel disease, including UC [18]. Recent studies from our laboratory have demonstrated that aldo-keto reductase 1B10 (AKR1B10) is a potential etiopathogenic factor of UC and CAC [19]. Among these etiopathological factors, the abnormal immune response is considered a key of UC. The normal colon mucosa plays an immune, endocrine, and barrier function. Injuries occurring in the intestinal mucosa insult its barrier function; increased intestinal mucosal permeability allows microbes and antigens to invade and excessively stimulate immune response, triggering intestinal inflammation. Excessive ROS are produced leading to oxidative stress during the inflammatory response, exaggerating inflammatory lesions in the pathogenesis of UC.

### 2.1. Redox in the Intestine

In the intestine, main ROS include hydroxyl free radical (OH^∙^), superoxide anion radical (O_2_^∙−^), and hydrogen peroxide (H_2_O_2_) while superoxide dismutase (SOD), glutathione peroxidase (GSH-PX), and catalase (CAT) are main antioxidant enzymes [20]. Peroxisomes are important organelles in biological oxidation in cells and participate in production and clearance of free radicals. Peroxisomes are enriched with hydrogen peroxide enzymes, oxidases, and peroxidases [21, 22]. Oxidases catalyze *β*-oxidation of fatty acids for energetic metabolism, producing H_2_O_2_. H_2_O_2_ is in turn transformed into OH^∙^ or other active free radicals [23]. In addition, xanthine oxidase and uric acid oxidase produce electronics in oxidative metabolic pathways [24, 25]. Hydrogen peroxide enzyme and SOD reduce H_2_O_2_ into H_2_O. In the process of chronic intestinal inflammation, a large amount of ROS, such as O_2_^∙−^ and H_2_O_2_, is produced by neutrophils during phagocytosis. This phagocytic process activates nicotinamide adenine dinucleotide phosphate oxidase (NOX) in the membrane, leading to rapid depletion of oxygen and production of superoxide anion [5, 6].

Cells evolve an antioxidant defense system to maintain homeostasis between the oxidant and antioxidant species [26, 27]. Excessive generation of free radicals beyond the capacity of defense leads to failure of this homeostatic process and oxidative injuries, such as lipid peroxidation and DNA damage, so-called oxidative stress. The antioxidant defense system in cells consists of enzymatic and nonenzymatic antioxidant molecules (Table 1). In addition to the endogenous cellular antioxidant species, natural food is also an important resource of antioxidants. For example, quercetin (3,5,7,3′,4′, pentahydroxyflavone), a flavonoid present in numerous fruits and vegetables, demonstrates appreciable antioxidant activity by eliminating free radicals and quenching singlet oxygen [28]. Resveratrol, a phenolic substance in red wines, is also a natural antioxidant and anti-inflammatory molecule [29].

### 2.2. Oxidative Stress Insults in Ulcerative Colitis

**Figure 1:**
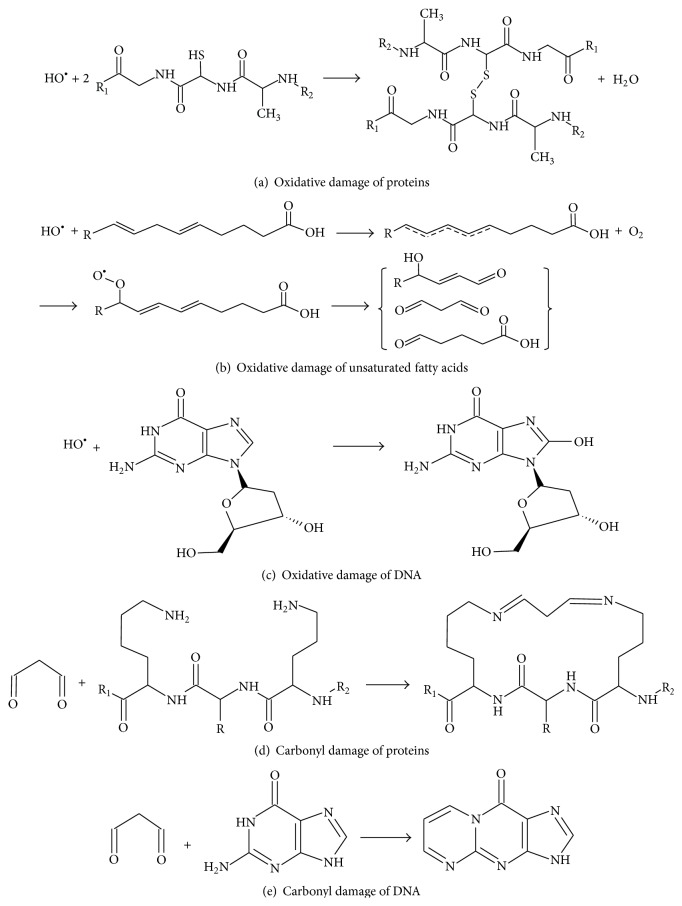
Schematic formulas. Reactive oxygen species and carbonyl compounds are highly reactive, causing protein, lipids, and DNA damage.

While a basal level of ROS may play a protective role in the intestine, the oxidative stress derived from imbalance between ROS production and antioxidant system is harmful, being an important pathogenic factor of UC. ROS are highly active chemical forms that target macromolecules, such as proteins, lipids, and nucleic acids, leading to lipid peroxidation, protein dysfunction, and DNA mutations (Figure 1). Therefore, excessive ROS cause cell and tissue damage, exaggerate inflammation, and lead to far-reaching effects, such as carcinogenesis. Herein we will discuss the protein and lipid damage and cellular effects induced by oxidative stress. Nuclei acid damage and carcinogenic effects of oxidative stress will be addressed in Section 3.

#### 2.2.1. Protein Damage Induced by Oxidative Stress

Oxidative stress insults proteins. Highly active ROS can readily interact with protein amino acid residues, such as His, Pro, Trp, Cys, and Tyr residues, and cause protein structure changes, polypeptide chain cracking, and loss of the biological activity. For example, ROS can oxidize sulfhydryl groups (-SH) in the amino acid residues to form disulfide bonds (-S-S-) and cross-link (Figure 1). ROS could also attack the methyl-sulfide group (CH_3_-S-) in methionine (Met) and affect hydrolysis and carbonylation of proteins [30, 31].

#### 2.2.2. Lipid Peroxidation Triggered by Oxidative Stress

Lipid peroxidation (LPO) is a serious cellular damage. Reactive ROS readily bind to unsaturated fatty acids in lipids that contain multiple double bonds, “steal” electrons, and trigger a free radical chain reaction (Figure 1). This oxidative process usually consists of initiation (production of a fatty acid radical), propagation (creation of a peroxyl-fatty acid radical), and termination (production of electrophilic carbonyls) [32]. This lipid peroxidation process produces two major biological effects, that is, direct membrane damage and permeability and production of lipid peroxides [33]. The common lipid peroxides created by lipid peroxidation include malondialdehyde (MAD), 4-hydroxynonenal (HNE), crotonaldehyde, and acrolein [34]. These lipid peroxides are *α*,*β*-unsaturated and highly reactive to cellular proteins and nucleic acids. In UC pathogenesis, lipid peroxides are important secondary injury factors of oxidative stress.

Phospholipids are primary ingredients of cell and organelle membrane and are enriched with unsaturated fatty acids. Therefore, the lipid peroxidation induced by oxidative stress mainly occurs in the membrane, and attacking by ROS would lead to direct structural and functional changes of membranes [33]. Mitochondrial membrane is the site of the respiratory chain that generates ROS in the normal cells. Therefore, mitochondria are the main organelles that are produced and attacked by ROS [35]. In the status of oxidative stress, excessive ROS attack oxidation respiratory chain and lead to obstacle of oxidative phosphorylation, producing more ROS. Excessive ROS also make Ca^2+^ overload in the mitochondria and lead to mitochondrial membrane depolarization and permeability, releasing free radicals into cytoplasm and causing cellular damage in general. Increased membrane permeability also releases cytochrome C (Cyt-C) and apoptosis inducing factor (AIF) into cytoplasm and activates caspase cascade for apoptosis [36, 37]. Therefore, in oxidative status ROS production by respiratory chain, mitochondrial membrane insults, and ROS release into cytoplasm form a vicious cycle, causing cell death and tissue injury. We will discuss the lesions induced by lipid peroxides in Section 2.3.

#### 2.2.3. Cell Signaling Triggered by Oxidative Stress

**Figure 2:**
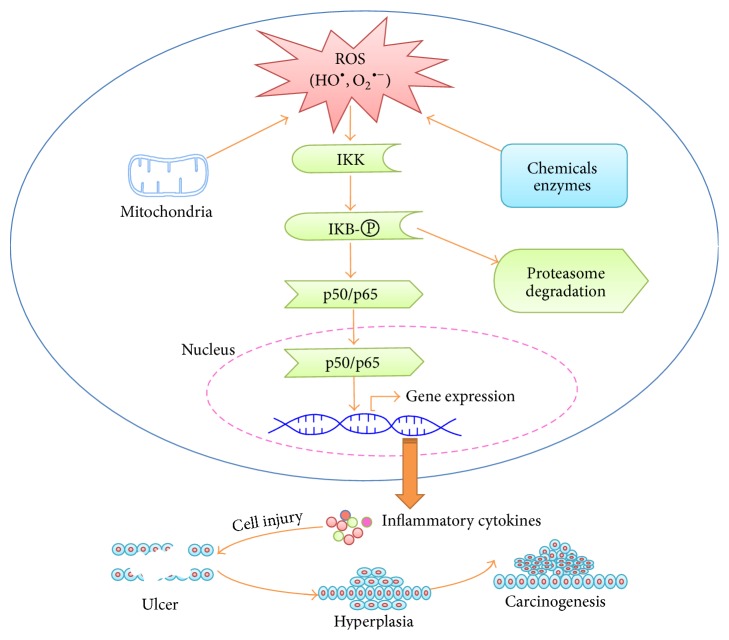
NF-*κ*B signaling pathway, inflammation, and carcinogenesis induced ROS. Excessive reactive oxygen species (ROS) derived from mitochondrial membrane, xenobiotics, and enzyme reactions activate IKK. Activated IKK phosphorylates I*κ*B and leads to ubiquitination and proteasome degradation of I*κ*B, releasing NF-*κ*B proteins, such as p50 and p65. The free p50 and p65 translocate into nuclei and drive target gene expression, such as inflammatory cytokines, leading to inflammatory lesions and carcinogenesis.

ROS could function as second messengers to activate intracellular signaling pathways, such as NF-*κ*B, a major modulator of UC [38–42]. In the normal intestinal epithelium, NF-*κ*B maintains intestinal epithelial barrier function and coordinates epithelial immune response to microorganisms. On the other hand, as transcription factors, deregulation of NF-*κ*B signaling, such as oxidative activation, stimulates expression of a variety of proinflammatory cytokines in the intestinal epithelial cells, such as TNF-*α*, IL-1, IL-8, and COX-2, and promotes inflammation and carcinogenesis. In static state, NF-*κ*B in the cells is bound to I*κ*B, inhibitors of *κ*B, and hooked in the cytoplasm. Activation of NF-*κ*B consists of I*κ*B kinase (IKK) activation, I*κ*B phosphorylation and ubiquitinated degradation by 26S proteasomes, and nuclear translocation and DNA binding of free NF-*κ*B, finally promoting target gene expression [43]. Oxidative stress can activate IKK and stimulate nuclear translocation of NF-*κ*B (Figure 2). In the diseased colon tissues of UC patients, NF-*κ*B expression, particularly the p65 (Re1A) and p52/p100 (NF-*κ*B2), is increased, and blockade of NF-*κ*B activity is considered practical treatment of UC [44]. In addition, the activation of p50, c-Rel, and p65 is documented in macrophages in the lamina propria of UC patients [45].

Oxidative stress also activates mitogen-activated protein (MAP) kinase (MAPK) signaling pathways. MAPKs are highly conserved serine/threonine protein kinases functioning in various fundamental cellular processes, such as growth/proliferation, differentiation, motility, and apoptosis/survival, as well as stress response [46]. Conventional MAPKs include the extracellular signal-regulated kinases 1 and 2 (Erk1/2), the c-Jun N-terminal kinases 1–3 (JNK1–3)/stress activated protein kinases (SAPK), the p38 isoforms (p38*α*, *β*, *γ*, and *δ*), and the Erk5. These MAPKs can be activated by growth factors and mitogens, as well as various stresses. These stimuli activate MAPKK kinases (MAPKKKs) via receptor dependent and independent mechanisms, followed by phosphorylation and activation of a downstream MAPK kinase (MAPKK) and then MAPKs. Activated MAPKs phosphorylate and activate specific target protein kinases, such as RSK, MSK, or MNK to mediate biological processes [47]. The increased ROS can activate ERKs, JNKs, or p38 MAPKs [48, 49]. The exact mechanism by which the ROS activate these kinases is unclear, but a plausible mechanism may be relative to oxidative modifications and resultant activation of the signaling effector proteins and inactivation and/or degradation of MAPK phosphatases (see [50] for more details). Nevertheless, the p38 and JNK signaling activated by ROS is involved in the disease progression of UC [51–54]. In UC tissues, p38 MAPK signaling changes are a molecular signature of UC and proportional to the degree of inflammation [55, 56].

### 2.3. Carbonyl Stress and a Vicious Cell Damage Cycle

A class of carbonyl compounds is called *α*,*β*-unsaturated carbonyls, also referred to as electrophilic carbonyls. These include acrolein, glyoxal, methylglyoxal, crotonaldehyde, malondialdehyde, and 4-hydroxynonenal (Table 2). As byproducts, these electrophilic carbonyl compounds are constantly produced during the metabolism of lipids, carbohydrates, amino acids, biogenic amines, vitamins, and steroids, as well as some antitumor agents, such as cyclophosphamide [57–63]. Besides endogenous production, daily food consumption may represent the most dangerous exposure of human gastrointestinal (GI) tract to exogenous electrophilic carbonyls which are pervasively present in various beverages and foodstuffs [64–66]. For instance, humans are exposed to crotonaldehyde through the consumption of vegetables (1.4–100 *µ*g/kg), fruits (5.4–78 *µ*g/kg), fish (71.4–1000 *µ*g/kg), meat (10–270 *µ*g/kg), and alcoholic beverages, such as wine (300–700 *µ*g/L) and whisky (30–210 *µ*g/L) [66]. Furthermore, methylglyoxal is a constituent of coffee [67, 68], and acetaldehyde is a carcinogenic metabolite of alcohol consumed [69, 70]. Therefore, human GI tract is repeatedly exposed to carbonyl threats, which are important factors of GI inflammatory and neoplastic lesions (Table 3).

In organisms, there are three main pathways responsible for elimination of intracellular carbonyls, through which carbonyls are oxidized to carbonic acids, conjugated with glutathione, or reduced to less toxic alcohols. Aldehyde dehydrogenases mediate the oxidative pathway of carbonyls, forming carbonic acids [71, 72]; glutathione-*S*-transferases (GST) catalyze the conjugation of carbonyls with glutathione [73–75]; and aldehyde reductase and aldo-keto reductases (AKRs) are responsible for the reduction of carbonyls to alcohols with NAD(P)H as a coenzyme [75–77]. AKR1B10 is the sole carbonyl-detoxifying enzyme with intestine-specific expression identified thus far [78] and plays a critical role in the inflammatory lesions and malignant progression of the colon [19]. Therefore, in normal conditions human consumption or endogenous production of the cytotoxic carbonyls may be subcytotoxic. However, in oxidative stress, excessive ROS oxidize unsaturated fatty acids and produce a large amount of highly reactive *α*,*β*-unsaturated carbonyl compounds, that is, lipid peroxides. For instance, 4-hydroxynonenal (HNE) is at 0.1 to 3.0 *µ*M in normal tissues but increases to ~10 *µ*M in the condition of oxidative stress [79]. Carbonyl accumulation due to overproduction and/or impaired clearance, such as AKR1B10 deficiency [19], would lead to carbonyl stress.

Due to their high reactivity, *α*,*β*-unsaturated lipid peroxides are highly cytotoxic and genotoxic. They can interact with free amino groups of proteins (e.g., lysine residue), peptides, and amino acids, with sulfhydryl groups of amino acid residues (e.g., cysteine residue), and with histidine and other residues, forming covalently modified adducts [57, 80–86]. The covalent modifications could lead to protein dysfunction, resistance to proteolysis, or depolymerization. Protein adducts can also act as special second messengers or autoantigens, promoting macrophage accumulation, retention, and activation, thus increasing ROS generation. Furthermore, carbonyl-induced protein dysfunction may impair mitochondrial respiratory chain reactions and membrane potential, leading to increased ROS production and release into cytosol. Therefore, in inflammatory conditions (i.e., UC), the carbonyl lesions may create a vicious loop with oxidative stress, aggravating cell and tissue damage [19, 87].

## 3. Oxidative Stress and Carbonyl Lesions in Colitis-Associated Colorectal Cancer

Colorectal cancer (CRC) is the third most common cancer worldwide with mortality ranked within top four [88, 89]. According to International Agency for Research on Cancer of WHO (http://globocan.iarc.fr/Pages/fact_sheets_cancer.aspx), about 1.36 million of new CRC cases were diagnosed globally in 2012, accounting for approximately 69,000 deaths. Clinically, there are two main types of CRC, that is, sporadic colorectal cancer (SCC) and hereditary colorectal cancer (HCC). The latter includes familial adenomatous polyposis (FAP) and hereditary nonpolyposis colorectal cancer (HNPCC). Colorectal adenoma, colorectal nonadenomatous polyposis, and inflammatory bowel disease are precancerous lesions associated with CRC. The UC patients have an increased risk of developing colorectal cancer, so-called colitis-associated colorectal cancer (CAC) [90], and the cancer risk increases exponentially with the duration of disease [91–93]. A UC patient with 10 years of disease duration has 10-fold higher CRC risk than the general population.

Etiopathogenesis of CAC is complex. In UC, intestinal epithelial and immune cells produce and secrete a variety of mitogenic cytokines that stimulate cell growth and proliferation. Massive ROS and inflammatory cytokines produced in UC tissues activate multiple signal pathways, such as NF-*κ*B, STAT3, p38 MAPK, and Wnt/*β*-catenin pathways, which mediate cell proliferation, differentiation, and apoptosis/survival [94]. Finally, DNA damage induced by oxidative and carbonyl stresses plays an essential role in the carcinogenic transformation of the disease. Therefore, malignant progression of UC to CAC is a complicated process and oxidative and carbonyl stresses are key factors in this process.

### 3.1. Sporadic Colorectal Cancer and Colitis-Associated Colorectal Cancer

CRC is a multistaged, complicated disease associated with multiple oncogene and tumor suppressor gene mutations, such as* p53, K-ras,* and adenomatous polyposis coli (*APC*) mutations [95]. In pathogenesis, sporadic CRC often demonstrates an “adenoma-carcinoma” progression, but the CAC experiences a unique sequence of “inflammation-dysplasia-carcinoma” [96]. Patients with UC may experience a long course of dysplasia. Three types of atypical hyperplasia may appear in the carcinogenic process of UC: (1) normal mucosa or mucous membrane with regeneration, also named dysplasia negative type, (2) dysplasia uncertain type, (3) dysplasia positive type. UC patients with high or moderate grade dysplasia are at high risk of developing CAC [97].

CAC also demonstrates a different time line and involvement of gene mutations. In sharp contrast to sporadic CRC,* p53* mutation occurs early and is an important step in the progression of CAC. The* p53* mutations are often detected in mucosa that is even nondysplastic [98, 99], but APC mutations are present at the late stage of CAC [100–103].* K*-*ras* mutation plays a rare role in CAC development [104], but DNA methylation is an early event in UC [105], although less common than in sporadic CRC [106, 107].

### 3.2. Inflammatory Cytokines and CAC Progression

Inflammatory cytokines produced by intestinal epithelial cells and infiltrated inflammatory cells in UC include IL-1, IL-6, TNF-*α*, and TGF-*β*. These cytokines activate mitogenic signaling pathways, stimulate cell proliferation and survival, and thus promote inflammation-associated tumorigenesis. For instance, the plasma level of IL-6 is significantly elevated in patients with IBD, and the increased IL-6 activates STAT3/JAKl signaling, promoting cell proliferation, evolution, and tumorigenic progression [94]; inhibition of JAKl signaling or IL-6 deficiency by targeted disruption diminishes CRC incidence and progression [108, 109].

Tumor necrosis factors (TNF) are proinflammatory cytokines which are produced and secreted mainly by monocyte-macrophages. In this family, TNF-*α* is an important member that functions in inflammation, immune response, and tumorigenesis. Animal experiments have demonstrated that TNF-*α* can increase the plasma level of IL-6 [110] and initiate colorectal carcinogenesis mediated by chronic inflammation [111]. To date, TNF-*α* monoclonal antibody is used for IBD treatment and has demonstrated promising results; this antibody may also be effective in prevention of CAC [112].

TGF-*β* and family members are secretory signal transduction peptides that regulate cell proliferation and apoptosis. In the normal cells, the major function of TGF-*β* is to arrest cell division in the early stage of DNA synthesis, induce cell differentiation, or promote apoptosis. Literature reports indicate that mutations in TGF-*β* signal transduction pathway occur in patients with UC before the formation of colorectal cancer [113]. For example, TGF-*β*RII mutations have been detected in UC dysplasia and are associated with CAC progression [114].

Finally, inflammatory cytokine IL-1*α* increases in UC and may be involved in CAC development [115], but compared to other cytokines, the role of IL-1*α* in the development and progression of CAC is more complicated. IL-1*α* may promote cancer progression by stimulating angiogenesis [116]; IL-1*α* may also promote epithelial repair and prevent CAC by inducing the expression of cyclooxygenase 2 (COX-2), a key enzyme of prostaglandin E_2_ (PGE_2_) synthesis from arachidonic acid (AA) [117]. PGE_2_ is a prominent prostaglandin in the intestine; through binding to E prostanoid (EP) receptor, PGE_2_ mediates intestinal epithelial cell proliferation and apoptosis [118, 119]. This is considered favorable to injury repair and remission of UC. In fact, ulcerogenic response of nonsteroidal anti-inflammatory drugs (NSAIDs) in the intestine is ascribed to inhibition of cyclooxygenases and resultant PGE_2_ deficiency [120]. In dextran sodium sulfate- (DSS-) induced colitis, COX-2/PGE_2_ promotes epithelial cell proliferation; inhibition of COX-2 decreases epithelial proliferation, exacerbates colitis, and prolongs injury phase, thus promoting intestinal injury and dysplasia [121–123]. Therefore, evaluation of IL-1*α* in CAC development and progression needs to be more cautious.

### 3.3. Oxidative DNA Damage in CAC Progression

DNA mutations and resultant protooncogene activation and/or tumor suppressor gene inactivation are a hallmark of cell carcinogenesis, which reprograms cell growth, division, and gene transcription. The high risk of UC patients to develop colorectal cancer is essentially attributed to the increased DNA damage induced by inflammatory oxidative stress and carbonyl lesions. DNA is a ready target of active oxygen free radicals, leading to oxidative DNA damage. Through abstractions and addition reactions, highly reactive hydroxyl radicals react with the heterocyclic DNA bases and sugar moiety, producing carbon-centered sugar radicals and OH- or H-adduct radicals of heterocyclic bases [124]. Further reactions of these radicals yield numerous effects, such as 8,5′-cyclopurine-2′-deoxynucleosides, tandem lesions, clustered sites, and DNA-protein cross-links [124, 125]. Among types of oxidative DNA damage induced by ROS, 8-hydroxy-2′-deoxyguanosine (8-OHdG) or 8-oxo-7,8-dihydro-2′-deoxyguanosine (8-oxodG) is a predominant form and a valuable biomarker widely used for endogenous oxidative damage to DNA (Figure 1). For instance, the urinary 8-OHdG is used as a biomarker for risk assessment of cancers and degenerative diseases [126, 127].

GC to TA transversion is a major type of DNA mutations resulting from 8-OHdG adducts [128]; two common target genes of the 8-OHdG damage are* Ras* and* p53*, leading to activation of the protooncogene* Ras* and inactivation of p53 tumor suppressor, driving tumorigenesis [129, 130]. ROS also cause DNA methylation, single- and double-strand breaks, and shortening of telomeres. DNA methylation is an early event in the progression of UC to CAC [105], but less common than in sporadic CRC [106, 107]. Oppositely, DNA breaks and telomere shortening occur more often in the UC-associated tumorigenesis [131, 132]. The telomere shortening induced by ROS could induce chromosome instability, leading to chromosomal loss, heteroploid, amplification, and translocation, driving tumorigenesis [133, 134].

### 3.4. Carbonyl DNA Damage in CAC Progression

Carbonyl stress derived from lipid peroxidation is also an important DNA damage factor in UC. Electrophilic carbonyls can readily react with DNA forming covalently modified DNA adducts (Figure 1). The DNA adducts can block DNA semiconservative replication performed by DNA polymerases or arrest transcription driven by RNA polymerases [58, 135–137]. DNA adducts can also cause miscoding and induce DNA breaks [58, 137–139]. For instance, malondialdehyde (MDA) can react with deoxyguanosine in DNA to form an exocyclic adduct, pyrimido[1,2-alpha]purin-10(3H)-one (M1G), which is mutagenic by resulting in frameshift mutations and base pair substitutions [140]. The 4-HNE-dG polymer derived from 4-hydroxynonenal can lead to GC to TA transversion at codon 249 of* p53* gene, driving UC progression to CAC [141, 142].

**Figure 3:**
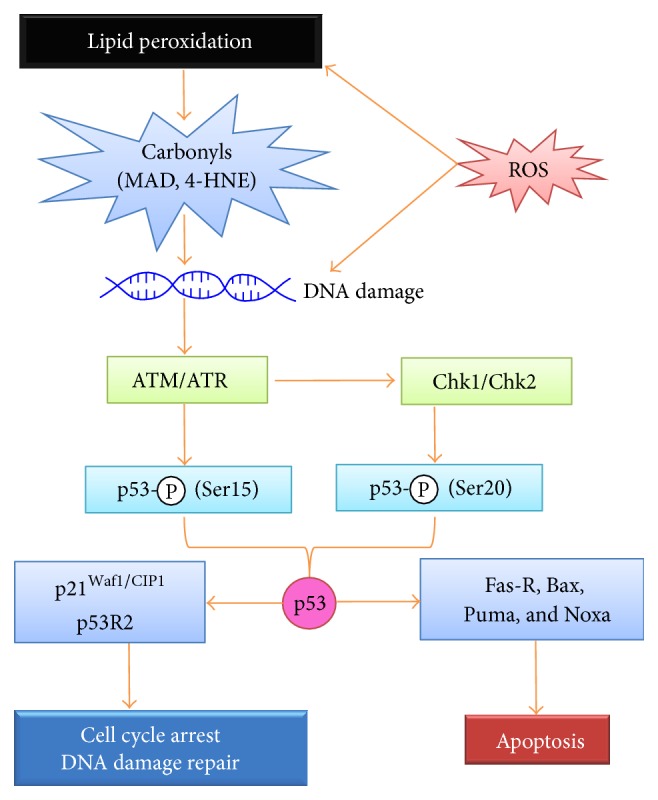
DNA damage induced by oxidative and carbonyl stresses and p53-dependent DNA damage response (DDR). Reactive oxygen species (ROS) and *α*,*β*-unsaturated carbonyl compounds produced by lipid peroxidation, such as MDA and HNE, trigger DNA damage, such as double-strand DNA breaks. ATM/ATR senses the breaks and activates p53 by phosphorylating Ser15; ATM/ATR also phosphorylates Ser345 of Chk1/Chk2 and activates Chk1/Chk2, which further activates p53 by phosphorylating Ser20. In cells with mild DNA damage, p53 drives expression of p21^Waf1/CIP1^ and p53R2, leading to cell cycle arrest and DNA damage repair. In cells with severe DNA damage, p53 drives Fas-R, Bax, Puma, Noxa, Apaf-1, and Pidd expression, activating intrinsic and extrinsic apoptotic pathways.

Of note, DNA breaks induced by carbonyl compounds may activate cellular DNA damage response (DDR), inducing cell cycle arrest for DNA repair or apoptosis (Figure 3). In this DDR process, ATM/ATR functions as a sensor of DNA breaks, and p53 acts as a key mediator [143, 144]. Sensing the DNA double-strand breaks, ATM/ATR is activated by phosphorylation, which reaches the peak within 30 minutes [145]. The activated ATM/ATR phosphorylates p53 at Ser15 and/or Chk1/Chk2 at Ser345, and Chk1/Chk2 further phosphorylate p53 at Ser20 [146]. Activated p53 triggers cell cycle arrest for DNA damage repair or apoptosis to eliminate cells with severe DNA damage through selective activation of target gene expression, such as apoptotic genes Fas-R, Bax, Puma, and Noxa or cell cycle monitoring and DNA repair genes p21^Waf1/CIP1^ and p53R2 [147]. Therefore, DDR is considered a barrier of carcinogenesis, and mutations of genes in this pathway are carcinogenic. In fact, p53 mutation is an early event in CAC and occurs even in noncancerous UC tissues [148, 149].

## 4. Conclusion and Perspective

**Figure 4:**
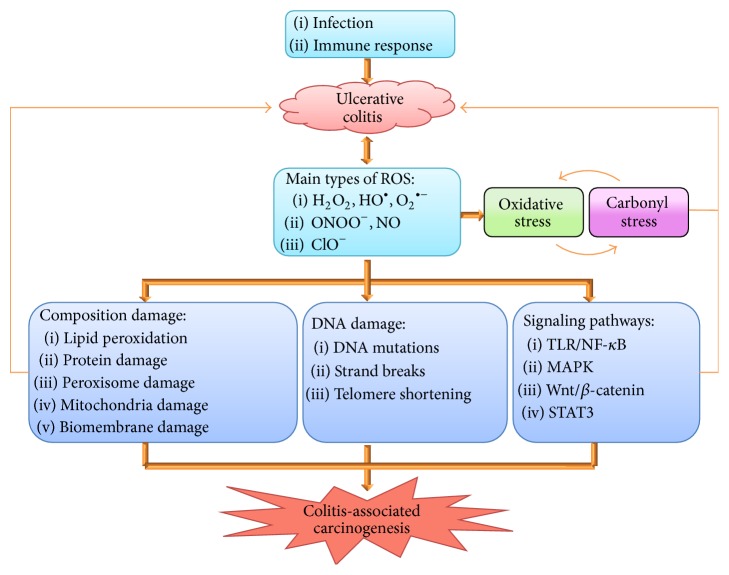
Hypothetic model of oxidative stress and carbonyl lesions in ulcerative colitis and associated colorectal cancer. Infection and immune response act as primary initiators to trigger inflammation and inflammatory cell infiltration. In this process, intestinal mucosal crypt abscesses occur and vast reactive oxygen species (ROS) are produced, thus leading to oxidative stress. Excessive ROS exaggerate inflammatory lesions and stimulate epithelial cell proliferation through oxidative insults to proteins, lipids, and DNA and also by activation of cell signaling pathways, eventually leading to ulcerative colitis (UC) and colitis-associated colorectal cancer (CAC). Electrophilic carbonyl compounds play as important secondary factors of oxidative stress to cause cellular and macromolecular lesions, which, together with oxidative stress, may form a vicious cycle. Meanwhile, proinflammatory cytokines produced by epithelial cells and infiltrated inflammatory cells may promote the progression of UC and CAC.

Early in 1863, a German pathologist Virchow proposed that tumor might be derived from chronic inflammation tissues; in 2009, Hanahan and Weinberg proposed tumor-related inflammation as the seventh hallmark of cancer. To date, the role of chronic inflammation in cancer development and progression has become an important research focus in tumor microenvironment. In UC, the pathogenesis of CAC is a classical path of nonresolving inflammatory progression to cancer, featured with a unique sequence of “inflammation-dysplasia-carcinoma.” Oxidative stress and secondary carbonyl lesions are key factors in the development and progression of UC and CAC; the ROS take an important part in multiple stages of initiation, promotion, and progression of UC and CAC and the secondary carbonyl lesions play an exaggerating role both in oxidative stress itself and in progression of UC and CAC (Figure 4).

To date, antioxidant prevention and treatment have been investigated in experimental animals of colitis and in clinical patients of UC. In animals, antioxidant* G. biloba* extract (EGb 761) showed effectiveness in prevention and treatment of DSS-induced colitis in mice [150], and the* Zingiber officinale* extract demonstrated efficacy in modulating extent and severity of colitis in rats [151]. In humans, consumptions of antioxidant food, such as blueberries, cherries, tomatoes, squashes, and bell peppers have been suggested as supplementary treatment of active UC and prevention of reactivation. More impressively, a clinical trial of rectal d-alpha tocopherol, a powerful vitamin E antioxidant, has shown that “all 14 patients responded clinically to the therapy and remission was induced in 9 of them (64%)”; no adverse events were reported and no patients were hospitalized for “worsened disease activity” [152]. These preclinical and clinical approaches suggest that antioxidant treatment may be a novel mode of UC management and prevention of malignant progression. Further studies are warranted.

## Figures and Tables

**Table 1:** Oxidant species and antioxidant defense.

Oxidant species	
Reactive oxygen species (ROS)	Superoxide anion (^∙^O_2_^−^)
	Peroxide (O_2_^−2^)
	Hydrogen peroxide (H_2_O_2_)
	Hydroxyl radical (^∙^OH)
	Hydroxyl ion (OH^−^)

Reactive nitrogen species (RNS)	Nitric oxide (^∙^NO)
	Peroxynitrite (ONOO^−^)
	Nitrogen dioxide (^∙^NO_2_)
	Dinitrogen trioxide (N_2_O_3_)
	Nitrosoperoxycarbonate (ONOOCO_2_^−^)

ROS resources	Mitochondrial electron transition
	Enzyme reactions: peroxisomal oxidases, cytochrome P-450, NAD(P)H oxidases, and xanthine oxidase
	Xenobiotics, drugs, and radiation: cisplatin, doxorubicin, and so forth.

RNS resources	Nitric oxide (^∙^NO) from nitric oxide synthase 2 (NOS2)
	Other RNS from reaction of nitric oxide (^∙^NO) with superoxide anion (^∙^O_2_^−^) and other reactive species.

Antioxidant defense	

Nonenzymatic antioxidants	Glutathione (GSH)
	Cysteine
	Metallothionein
	Coenzyme Q (CoQ)
	Uric acid

Enzymatic antioxidants	Superoxide dismutase (SOD)
	Catalase (CAT)
	Peroxiredoxin (Prx)
	Glutathione reductase (GR)
	Glutathione peroxidases (GPx)
	Glutathione-S-transferases (GST)
	Thioredoxin and thioredoxin reductase

Natural (food) antioxidants	Vitamin A/C/E
	Flavonoid
	Resveratrol

**Table 2:** Carbonyl compounds and clearance.

Carbonyl compounds	Carbonyl clearance
Acrolein (CH_2_=CHCHO) Glyoxal (OHCCHO) Methylglyoxal (CH_3_COCHO) Crotonaldehyde (CH_3_CH=CHCHO) Malondialdehyde (OCHCH_2_CHO) 4-Hydroxynonenal (OCHCH=CHCH(-OH)(CH_2_)_4_CH_3_)	(1) Glutathione-*S*-transferases (GST) catalyze carbonyl-glutathione conjugation (2) Aldehyde reductase and aldo-keto reductases (AKRs) catalyze reduction to alcoholic forms (3) Aldehyde dehydrogenases catalyze oxidation to carbonic acids

**Table 3:** Carcinogenic role of carbonyl compounds.

Diseases/genotoxicity	Species	Carbonyl association	References
Colitis-associated colorectal neoplasms	Mice	Coupled with high carbonyl levels, for example, malondialdehyde	[19, 153]

Stomach hyperplasia, squamous papilloma, and carcinoma	Rats	2,4-Hexadienal exposure	[73]

Precancerous gastritis and gastric cancer	Humans	High serum malondialdehyde levels	[154, 155]

Colorectal cancer	Humans	High serum lipid peroxide levels	[156]

Colon and gastric cancers	Humans	Acetaldehyde from alcohol	[69, 70]

Colorectal adenocarcinoma	Humans	High protein carbonyl levels	[157]

Precancerous colorectal adenopolyps	Humans	High protein carbonyl levels	[158]

Colorectal cancer	Humans	High lipid peroxide levels in tissues	[159–161]

Genotoxicity	Humans	High carbonyl DNA adduct levels in tissues	[58, 162, 163]

Genotoxicity	Cell lines/*in vitro* studies	Production of carbonyl DNA adducts	[164–167]

## Abbreviations

AIF: Apoptosis inducing factor
AKR1B10: Aldo-keto reductase 1B10
APC: Adenomatous polyposis coli
CAC: Colitis-associated colorectal cancer
CAT: Catalase
COX-2: Cyclooxygenase 2
CRC: Colorectal cancer
DSS: Dextran sodium sulfate
GSH-PX: Glutathione peroxidase
IBD: Inflammatory bowel disease
MAD: Malondialdehyde
NOX: Nicotinamide adenine dinucleotide phosphate oxidase
OS: Oxidative stress
Raf: Root abundant factor
ROS: Reactive oxygen species
SCC: Sporadic colorectal cancer
SOD: Superoxide dismutase
TGF-*β*: Transforming growth factor *β*
TNF-*α*: Tumor necrosis factor *α*
UC: Ulcerative stress.

## References

[1] D'Autréaux B., Toledano M. B. (2007). ROS as signalling molecules: mechanisms that generate specificity in ROS homeostasis. *Nature Reviews Molecular Cell Biology*.

[2] Lambert G. P. (2009). Stress-induced gastrointestinal barrier dysfunction and its inflammatory effects. *Journal of animal science*.

[3] Karp S. M., Koch T. R. (2006). Oxidative stress and antioxidants in inflammatory bowel disease. *Disease-A-Month*.

[4] Cheeseman K. H., Slater T. F. (1993). An introduction to free radical biochemistry. *British Medical Bulletin*.

[5] Geiszt M., Lekstrom K., Brenner S. (2003). NAD(P)H oxidase 1, a product of differentiated colon epithelial cells, can partially replace glycoprotein 91phox in the regulated production of superoxide by phagocytes. *Journal of Immunology*.

[6] Dutta S., Rittinger K. (2010). Regulation of NOXO1 activity through reversible interactions with p22phox and NOXA1. *PLoS ONE*.

[7] Baumgart D. C., Dignass A. U. (2002). Intestinal barrier function. *Current Opinion in Clinical Nutrition and Metabolic Care*.

[8] Okur H., Küçükaydin M., Köse K., Kontaş O., Doğan P., Kazez A. (1995). Hypoxia-induced necrotizing enterocolitis in the immature rat: the role of lipid peroxidation and management by vitamin E. *Journal of Pediatric Surgery*.

[9] Chan K. L., Hui C. W. C., Chan K. W. (2002). Revisiting ischemia and reperfusion injury as a possible cause of necrotizing enterocolitis: role of nitric oxide and superoxide dismutase. *Journal of Pediatric Surgery*.

[10] Danese S., Fiocchi C. (2011). Ulcerative colitis. *The New England Journal of Medicine*.

[11] Irving P. M. (2009). Recent advances in the management of inflammatory bowel disease. *Clinical Medicine*.

[12] Meier J., Sturm A. (2011). Current treatment of ulcerative colitis. *World Journal of Gastroenterology*.

[13] Weiss U. (2008). Inflammation. *Nature*.

[14] Bitton A., Peppercorn M. A., Antonioli D. A. (2001). Clinical, biological, and histologic parameters as predictors of relapse in ulcerative colitis. *Gastroenterology*.

[15] Oshitani N., Sawa Y., Hara J. (1997). Functional and phenotypical activation of leucocytes in inflamed human colonic mucosa. *Journal of Gastroenterology and Hepatology*.

[16] Podolsky D. K. (2002). Inflammatory bowel disease. *The New England Journal of Medicine*.

[17] Head K. A., Jurenka J. J. S. (2003). Inflammatory bowel disease part I: ulcerative colitis—pathophysiology and conventional and alternative treatment options. *Alternative Medicine Review*.

[18] Satsangi J., Parkes M., Louis E. (1996). Two stage genome-wide search in inflammatory bowel disease provides evidence for susceptibility loci on chromosomes 3, 7 and 12. *Nature Genetics*.

[19] Shen Y., Ma J., Yan R. (2015). Impaired Self-Renewal and Increased Colitis and Dysplastic Lesions in Colonic Mucosa of AKR1B8-Deficient Mice. *Clinical Cancer Research*.

[20] Zhu H., Li Y. R. (2012). Oxidative stress and redox signaling mechanisms of inflammatory bowel disease: updated experimental and clinical evidence. *Experimental Biology and Medicine*.

[21] Magliano P., Flipphi M., Arpat B. A., Delessert S., Poirier Y. (2011). Contributions of the peroxisome and *β*-oxidation cycle to biotin synthesis in fungi. *The Journal of Biological Chemistry*.

[22] Gehrmann W., Elsner M. (2011). A specific fluorescence probe for hydrogen peroxide detection in peroxisomes. *Free Radical Research*.

[23] Fransen M., Nordgren M., Wang B., Apanasets O. (2012). Role of peroxisomes in ROS/RNS-metabolism: implications for human disease. *Biochimica et Biophysica Acta—Molecular Basis of Disease*.

[24] Engerson T. D., McKelvey G., Rhyne D. B., Boggio E. B., Snyder S. J., Jones H. P. (1987). Conversion of xanthine dehydrogenase to oxidase in ischemic rat tissues. *Journal of Clinical Investigation*.

[25] Angermuller S., Fahimi H. D. (1986). Ultrastructural cytochemical localization of uricase in peroxisomes of rat liver. *Journal of Histochemistry and Cytochemistry*.

[26] Cerutti P., Ghosh R., Oya Y., Amstad P. (1994). The role of the cellular antioxidant defense in oxidant carcinogenesis. *Environmental Health Perspectives*.

[27] Rahal A., Kumar A., Singh V. (2014). Oxidative stress, prooxidants, and antioxidants: the interplay. *BioMed Research International*.

[28] Lee J., Hahm E.-R., Singh S. V. (2010). Withaferin A inhibits activation of signal transducer and activator of transcription 3 in human breast cancer cells. *Carcinogenesis*.

[29] de La Lastra C. A., Villegas I. (2007). Resveratrol as an antioxidant and pro-oxidant agent: mechanisms and clinical implications. *Biochemical Society Transactions*.

[30] Brookes P., Darley-Usmar V. M. (2002). Hypothesis: the mitochondrial NO(^∗^) signaling pathway, and the transduction of nitrosative to oxidative cell signals: an alternative function for cytochrome C oxidase. *Free Radical Biology and Medicine*.

[31] Cai Z., Yan L. J. (2013). Protein oxidative modifications: beneficial roles in disease and health. *Journal of Biochemical and Pharmacological Research*.

[32] Hodges G. R., Ingold K. U. (2000). Superoxide, amine buffers and tetranitromethane: a novel free radical chain reaction. *Free Radical Research*.

[33] Sargis R. M., Subbaiah P. V. (2006). Protection of membrane cholesterol by sphingomyelin against free radical-mediated oxidation. *Free Radical Biology and Medicine*.

[34] Juránek I., Bezek Š. (2005). Controversy of free radical hypothesis: reactive oxygen species—cause or consequence of tissue injury. *General Physiology and Biophysics*.

[35] Calabrese V., Lodi R., Tonon C. (2005). Oxidative stress, mitochondrial dysfunction and cellular stress response in Friedreich's ataxia. *Journal of the Neurological Sciences*.

[36] Wasiak S., Zunino R., McBride H. M. (2007). Bax/Bak promote sumoylation of DRP1 and its stable association with mitochondria during apoptotic cell death. *The Journal of Cell Biology*.

[37] Paradies G., Petrosillo G., Paradies V., Ruggiero F. M. (2009). Role of cardiolipin peroxidation and Ca^2+^ in mitochondrial dysfunction and disease. *Cell Calcium*.

[38] Tak P. P., Firestein G. S. (2001). NF-kappaB: a key role in inflammatory diseases. *The Journal of Clinical Investigation*.

[39] Karrasch T., Jobin C. (2008). NF-*κ*B and the intestine: friend or foe?. *Inflammatory Bowel Diseases*.

[40] Cummins E. P., Doherty G. A., Taylor C. T. (2013). Hydroxylases as therapeutic targets in inflammatory bowel disease. *Laboratory Investigation*.

[41] Wang K., Grivennikov S. I., Karin M. (2013). Implications of anti-cytokine therapy in colorectal cancer and autoimmune diseases. *Annals of the Rheumatic Diseases*.

[42] Schreck R., Albermann K., Baeuerle P. A. (1992). Nuclear factor kb: an oxidative stress-responsive transcription factor of eukaryotic cells (a review). *Free Radical Research*.

[43] Pantano C., Reynaert N. L., van der Vliet A., Janssen-Heininger Y. M. W. (2006). Redox-sensitive kinases of the nuclear factor-*κ*B signaling pathway. *Antioxidants and Redox Signaling*.

[44] Baldwin A. S. (1996). The NF-*κ*B and I*κ*B proteins: new discoveries and insights. *Annual Review of Immunology*.

[45] Kaser A., Zeissig S., Blumberg R. S. (2010). Inflammatory bowel disease. *Annual Review of Immunology*.

[46] Topcu-Tarladacalisir Y., Akpolat M., Uz Y. H. (2013). Effects of curcumin on apoptosis and oxidoinflammatory regulation in a rat model of acetic acid-induced colitis: the roles of c-Jun N-terminal kinase and p38 mitogen-activated protein kinase. *Journal of Medicinal Food*.

[47] Higa A., Chevet E. (2012). Redox signaling loops in the unfolded protein response. *Cellular Signalling*.

[48] Waetzig G. H., Seegert D., Nikolaus S., Rosenstiel P., Sfikas N., Schreiber S. (2001). Differential activity and expression of mitogen-activated protein kinases in inflammatory bowel disease. *Gastroenterology*.

[49] Waetzig G. H., Seegert D., Rosenstiel P., Nikolaus S., Schreiber S. (2002). p38 mitogen-activated protein kinase is activated and linked to TNF-*α* signaling in inflammatory bowel disease. *Journal of Immunology*.

[50] Son Y., Cheong Y., Kim N., Chung H., Kang D. G., Pae H. (2011). Mitogen-activated protein kinases and reactive oxygen species: how can ROS activate MAPK pathways?. *Journal of Signal Transduction*.

[51] Hollenbach E., Neumann M., Vieth M., Roessner A., Malfertheiner P., Naumann M. (2004). Inhibition of p38 MAP kinase- and RICK/NF-*κ*B-signaling suppresses inflammatory bowel disease. *The FASEB Journal*.

[52] Hollenbach E., Vieth M., Roessner A., Neumann M., Malfertheiner P., Naumann M. (2005). Inhibition of RICK/nuclear factor-*κ*B and p38 signaling attenuates the inflammatory response in a murine model of Crohn disease. *Journal of Biological Chemistry*.

[53] Wei J., Feng J. (2010). Signaling pathways associated with inflammatory bowel disease. *Recent Patents on Inflammation and Allergy Drug Discovery*.

[54] Feng Y. J., Li Y. Y. (2011). The role of p38 mitogen-activated protein kinase in the pathogenesis of inflammatory bowel disease. *Journal of Digestive Diseases*.

[55] Hommes D., van den Blink B., Plasse T. (2002). Inhibition of stress-activated MAP kinases induces clinical improvement in moderate to severe Crohn's disease. *Gastroenterology*.

[56] Zhao X., Kang B., Lu C. (2011). Evaluation of P38 MAPK pathway as a molecular signature in ulcerative colitis. *Journal of Proteome Research*.

[57] Davydov V. V., Dobaeva N. M., Bozhkov A. I. (2004). Possible role of alteration of aldehyde's scavenger enzymes during aging. *Experimental Gerontology*.

[58] De Bont R., van Larebeke N. (2004). Endogenous DNA damage in humans: a review of quantitative data. *Mutagenesis*.

[59] Jain S. K., Mohandas N., Clark M. R., Shohet S. B. (1983). The effect of malonyldialdehyde, a product of lipid peroxidation, on the deformability, dehydration and ^51^Cr-survival of erythrocytes. *British Journal of Haematology*.

[60] Ullrich O., Henke W., Grune T., Siems W. G. (1996). The effect of the lipid peroxidation product 4-hydroxynonenal and of its metabolite 4-hydroxynonenoic acid on respiration of rat kidney cortex mitochondria. *Free Radical Research*.

[61] Choudhary S., Xiao T., Srivastava S. (2005). Toxicity and detoxification of lipid-derived aldehydes in cultured retinal pigmented epithelial cells. *Toxicology and Applied Pharmacology*.

[62] Berhane K., Widersten M., Engström Å., Kozarich J. W., Mannervik B. (1994). Detoxication of base propenals and other *α*,*β*-unsaturated aldehyde products of radical reactions and lipid peroxidation by human glutathione transferases. *Proceedings of the National Academy of Sciences of the United States of America*.

[63] Vaca C. E., Nilsson J. A., Fang J.-L., Grafström R. C. (1998). Formation of DNA adducts in human buccal epithelial cells exposed to acetaldehyde and methylglyoxal in vitro. *Chemico-Biological Interactions*.

[64] Ames B. N. (1983). Dietary carcinogens and anticarcinogens. Oxygen radicals and degenerative diseases. *Science*.

[65] Fujioka K., Shibamoto T. (2004). Formation of genotoxic dicarbonyl compounds in dietary oils upon oxidation. *Lipids*.

[66] Schuler B. D., Eder E. (2000). Development of a 32P-postlabelling method for the detection of 1,N2-propanodeoxyguanosine adducts of crotonaldehyde in vivo. *Archives of Toxicology*.

[67] Kasai H., Kumeno K., Yamaizumi Z. (1982). Mutagenicity of methylglyoxal in coffee. *Gann*.

[68] Nukaya H., Iwami T., Ishida H. (1990). N-2 Acetylation of 2′-deoxyguanosine by coffee mutagens, methylglyoxal and hydrogen peroxide. *Mutation Research Letters*.

[69] Homann N., Tillonen J., Salaspuro M. (2000). Microbially produced acetaldehyde from ethanol may increase the risk of colon cancer via folate deficiency. *International Journal of Cancer*.

[70] Salaspuro M. P. (2003). Alcohol consumption and cancer of the gastrointestinal tract. *Bailliere's Best Practice and Research in Clinical Gastroenterology*.

[71] Vasiliou V., Pappa A., Petersen D. R. (2000). Role of aldehyde dehydrogenases in endogenous and xenobiotic metabolism. *Chemico-Biological Interactions*.

[72] Sládek N. E. (2003). Human aldehyde dehydrogenases: potential pathological, pharmacological, and toxicological impact. *Journal of Biochemical and Molecular Toxicology*.

[73] Nyska A., Moomaw C. R., Lomnitski L., Chan P. C. (2001). Glutathione S-transferase Pi expression in forestomach carcinogenesis process induced by gavage-administered 2,4-hexadienal in the F344 rat. *Archives of Toxicology*.

[74] Coles B. F., Kadlubar F. F. (2003). Detoxification of electrophilic compounds by glutathione S-transferase catalysis: determinants of individual response to chemical carcinogens and chemotherapeutic drugs?. *BioFactors*.

[75] Srivastava S., Chandra A., Bhatnagar A., Srivastava S. K., Ansari N. H. (1995). Lipid peroxidation product, 4-hydroxynonenal and its conjugate with GSH are excellent substrates of bovine lens aldose reductase. *Biochemical and Biophysical Research Communications*.

[76] Zhong L., Liu Z., Yan R. (2009). Aldo-keto reductase family 1 B10 protein detoxifies dietary and lipid-derived alpha, beta-unsaturated carbonyls at physiological levels. *Biochemical and Biophysical Research Communications*.

[77] Shen Y., Zhong L., Johnson S., Cao D. (2011). Human aldo-keto reductases 1B1 and 1B10: a comparative study on their enzyme activity toward electrophilic carbonyl compounds. *Chemico-Biological Interactions*.

[78] Cao D., Fan S. T., Chung S. S. M. (1998). Identification and characterization of a novel human aldose reductase- like gene. *The Journal of Biological Chemistry*.

[79] Esterbauer H., Eckl P., Ortner A. (1990). Possible mutagens derived from lipids and lipid precursors. *Mutation Research/Reviews in Genetic Toxicology*.

[80] Li H., Wang J., Kaphalia B. S., Ansari G. A. S., Khan M. F. (2004). Quantitation of acrolein-protein adducts: potential biomarker of acrolein exposure. *Journal of Toxicology and Environmental Health Part: A*.

[81] Hashimoto M., Sibata T., Wasada H., Toyokuni S., Uchida K. (2003). Structural basis of protein-bound endogenous aldehydes. Chemical and immunochemical characterizations of configurational isomers of a 4-hydroxy-2-nonenal-histidine adduct. *The Journal of Biological Chemistry*.

[82] Uchida K., Stadtman E. R. (1992). Modification of histidine residues in proteins by reaction with 4- hydroxynonenal. *Proceedings of the National Academy of Sciences of the United States of America*.

[83] Alvarez J. G., Storey B. T. (1984). Assessment of cell damage caused by spontaneous lipid peroxidation in rabbit spermatozoa. *Biology of Reproduction*.

[84] Okada K., Wangpoengtrakul C., Osawa T., Toyokuni S., Tanaka K., Uchida K. (1999). 4-Hydroxy-2-nonenal-mediated impairment of intracellular proteolysis during oxidative stress. Identification of proteasomes as target molecules. *The Journal of Biological Chemistry*.

[85] Uchida K., Stadtman E. R. (1992). Selective cleavage of thioether linkage in proteins modified with 4-hydroxynonenal. *Proceedings of the National Academy of Sciences of the United States of America*.

[86] Hauptlorenz S., Esterbauer H., Moll W., Pümpel R., Schauenstein E., Puschendorf B. (1985). Effects of the lipidperoxidation product 4-hydroxynonenal and related aldehydes on proliferation and viability of cultured ehrlich ascites tumor cells. *Biochemical Pharmacology*.

[87] Coussens L. M., Werb Z. (2002). Inflammation and cancer. *Nature*.

[88] Cunningham D., Atkin W., Lenz H.-J. (2010). Colorectal cancer. *The Lancet*.

[89] Chawla N., Butler E. N., Lund J., Warren J. L., Harlan L. C., Yabroff K. R. (2013). Patterns of colorectal cancer care in Europe, Australia, and New Zealand. *Journal of the National Cancer Institute—Monographs*.

[90] Seril D. N., Liao J., Yang G.-Y., Yang C. S. (2003). Oxidative stress and ulcerative colitis-associated carcinogenesis: studies in humans and animal models. *Carcinogenesis*.

[91] Danese S., Malesci A., Vetrano S. (2011). Colitis-associated cancer: the dark side of inflammatory bowel disease. *Gut*.

[92] Collins R. H., Feldman M., Fordtran J. S. (1987). Colon cancer, dysplasia, and surveillance in patients with ulcerative colitis. A critical review. *The New England Journal of Medicine*.

[93] Ekbom A., Helmick C., Zack M., Adami H.-O. (1990). Ulcerative colitis and colorectal cancer. A population-based study. *The New England Journal of Medicine*.

[94] Grivennikov S., Karin E., Terzic J. (2009). IL-6 and Stat3 are required for survival of intestinal epithelial cells and development of colitis-associated cancer. *Cancer Cell*.

[95] Aretz S., Vasen H. F. A., Olschwang S. (2011). Clinical utility gene card for: familial adenomatous polyposis (FAP) and attenuated FAP (AFAP). *European Journal of Human Genetics*.

[96] Itzkowitz S. H., Yio X. (2004). Inflammation and cancer IV. Colorectal cancer in inflammatory bowel disease: the role of inflammation. *American Journal of Physiology—Gastrointestinal and Liver Physiology*.

[97] Lakatos L., Mester G., Erdelyi Z. (2006). Risk factors for ulcerative colitis-associated colorectal cancer in a Hungarian cohort of patients with ulcerative colitis: results of a population-based study. *Inflammatory Bowel Diseases*.

[98] Triantafillidis J. K., Nasioulas G., Kosmidis P. A. (2009). Colorectal cancer and inflammatory bowel disease: epidemiology, risk factors, mechanisms of carcinogenesis and prevention strategies. *Anticancer Research*.

[99] Ullman T. A., Itzkowitz S. H. (2011). Intestinal inflammation and cancer. *Gastroenterology*.

[100] Aust D. E., Terdiman J. P., Willenbucher R. F. (2002). The APC/*β*-catenin pathway in ulcerative colitis-related colorectal carcinomas: a mutational analysis. *Cancer*.

[101] You J., Nguyen A. V., Albers C. G., Lin F., Holcombe R. F. (2008). Wnt pathway-related gene expression in inflammatory bowel disease. *Digestive Diseases and Sciences*.

[102] Rapozo D. C. M., Grinmann A. B., Carvalho A. T. P. (2009). Analysis of mutations in TP53, APC, K-ras, and DCC genes in the non-dysplastic mucosa of patients with inflammatory bowel disease. *International Journal of Colorectal Disease*.

[103] Tarmin L., Yin J., Harpaz N. (1995). Adenomatous polyposis coli gene mutations in ulcerative colitis-associated dysplasias and cancers versus sporadic colon neoplasms. *Cancer Research*.

[104] Azer S. A. (2013). Overview of molecular pathways in inflammatory bowel disease associated with colorectal cancer development. *European Journal of Gastroenterology and Hepatology*.

[105] Wang F.-Y., Arisawa T., Tahara T. (2008). Aberrant DNA methylation in ulcerative colitis without neoplasia. *Hepato-Gastroenterology*.

[106] Konishi K., Shen L., Wang S., Meltzer S. J., Harpaz N., Issa J.-P. J. (2007). Rare CpG island methylator phenotype in ulcerative colitis-associated neoplasias. *Gastroenterology*.

[107] Sanchez J. A., Dejulius K. L., Bronner M., Church J. M., Kalady M. F. (2011). Relative role of methylator and tumor suppressor pathways in ulcerative colitis-associated colon cancer. *Inflammatory Bowel Diseases*.

[108] Lin W.-W., Karin M. (2007). A cytokine-mediated link between innate immunity, inflammation, and cancer. *The Journal of Clinical Investigation*.

[109] Becker C., Fantini M. C., Schramm C. (2004). TGF-*β* suppresses tumor progression in colon cancer by inhibition of IL-6 trans-signaling. *Immunity*.

[110] Anderson G. M., Nakada M. T., DeWitte M. (2004). Tumor necrosis factor-*α* in the pathogenesis and treatment of cancer. *Current Opinion in Pharmacology*.

[111] Popivanova B. K., Kitamura K., Wu Y. (2008). Blocking TNF-*α* in mice reduces colorectal carcinogenesis associated with chronic colitis. *Journal of Clinical Investigation*.

[112] Allez M., Vermeire S., Mozziconacci N. (2010). The efficacy and safety of a third anti-TNF monoclonal antibody in Crohn's disease after failure of two other anti-TNF antibodies. *Alimentary Pharmacology and Therapeutics*.

[113] Engle S. J., Ormsby I., Pawlowski S. (2002). Elimination of colon cancer in germ-free transforming growth factor beta 1-deficient mice. *Cancer Research*.

[114] Souza R. F., Lei J., Yin J. (1997). A transforming growth factor beta 1 receptor type II mutation in ulcerative colitis-associated neoplasms. *Gastroenterology*.

[115] Iwagaki H., Hizuta A., Tanaka N. (1997). Interleukin-1 receptor antagonists and other markers in colorectal cancer patients. *Scandinavian Journal of Gastroenterology*.

[116] Matsuo Y., Sawai H., Ma J. (2009). IL-1*α* secreted by colon cancer cells enhances angiogenesis: the relationship between IL-1*α* release and tumor cells' potential for liver metastasis. *Journal of Surgical Oncology*.

[117] Di Mari J. F., Saada J. I., Mifflin R. C., Valentich J. D., Powell D. W. (2007). HETEs enhance IL-1-mediated COX-2 expression via augmentation of message stability in human colonic myofibroblasts. *American Journal of Physiology: Gastrointestinal and Liver Physiology*.

[118] Stenson W. F. (2007). Prostaglandins and epithelial response to injury. *Current Opinion in Gastroenterology*.

[119] Hult L. T., Kleiveland C. R., Fosnes K., Jacobsen M., Lea T. (2011). EP receptor expression in human intestinal epithelium and localization relative to the stem cell zone of the crypts. *PLoS ONE*.

[120] Tanaka K.-I., Suemasu S., Ishihara T., Tasaka Y., Arai Y., Mizushima T. (2009). Inhibition of both COX-1 and COX-2 and resulting decrease in the level of prostaglandins E2 is responsible for non-steroidal anti-inflammatory drug (NSAID)-dependent exacerbation of colitis. *European Journal of Pharmacology*.

[121] Okayama M., Hayashi S., Aoi Y., Nishio H., Kato S., Takeuchi K. (2007). Aggravation by selective COX-1 and COX-2 inhibitors of dextran sulfate sodium (DSS)-induced colon lesions in rats. *Digestive Diseases and Sciences*.

[122] Tessner T. G., Cohn S. M., Schloemann S., Stenson W. F. (1998). Prostaglandins prevent decreased epithelial cell proliferation associated with dextran sodium sulfate injury in mice. *Gastroenterology*.

[123] Brown S. L., Riehl T. E., Walker M. R. (2007). Myd88-dependent positioning of Ptgs2-expressing stromal cells maintains colonic epithelial proliferation during injury. *Journal of Clinical Investigation*.

[124] Dizdaroglu M., Jaruga P., Birincioglu M., Rodriguez H. (2002). Free radical-induced damage to DNA: mechanisms and measurement. *Free Radical Biology and Medicine*.

[125] Kawanishi S., Hiraku Y., Pinlaor S., Ma N. (2006). Oxidative and nitrative DNA damage in animals and patients with inflammatory diseases in relation to inflammation-related carcinogenesis. *Biological Chemistry*.

[126] Kasai H. (1997). Analysis of a form of oxidative DNA damage, 8-hydroxy-2′-deoxyguanosine, as a marker of cellular oxidative stress during carcinogenesis. *Mutation Research - Reviews in Mutation Research*.

[127] Fortini P., Pascucci B., Parlanti E., D'Errico M., Simonelli V., Dogliotti E. (2003). 8-oxoguanine DNA damage: at the crossroad of alternative repair pathways. *Mutation Research*.

[128] Wang D., Kreutzer D. A., Essigmann J. M. (1998). Mutagenicity and repair of oxidative DNA damage: insights from studies using defined lesions. *Mutation Research: Fundamental and Molecular Mechanisms of Mutagenesis*.

[129] Calistri D., Rengucci C., Seymour I. (2005). Mutation analysis of p53, K-ras, and BRAF genes in colorectal cancer progression. *Journal of Cellular Physiology*.

[130] Solomon H., Brosh R., Buganim Y., Rotter V. (2010). Inactivation of the p53 tumor suppressor gene and activation of the Ras oncogene: cooperative events in tumorigenesis. *Discovery Medicine*.

[131] Kinouchi Y., Hiwatashi N., Chida M. (1998). Telomere shortening in the colonic mucosa of patients with ulcerative colitis. *Journal of Gastroenterology*.

[132] O'Sullivan J. N., Bronner M. P., Brentnall T. A. (2002). Chromosomal instability in ulcerative colitis is related to telomere shortening. *Nature Genetics*.

[133] Willenbucher R. F., Zelman S. J., Ferrell L. D., Moore D. H., Waldman F. M. (1997). Chromosomal alterations in ulcerative colitis-related neoplastic progression. *Gastroenterology*.

[134] Risques R. A., Lai L. A., Himmetoglu C. (2011). Ulcerative colitis-associated colorectal cancer arises in a field of short telomeres, senescence, and inflammation. *Cancer Research*.

[135] Nagy E., Zeisig M., Kawamura K. (2005). DNA adduct and tumor formations in rats after intratracheal administration of the urban air pollutant 3-nitrobenzanthrone. *Carcinogenesis*.

[136] Cline S. D., Riggins J. N., Tornaletti S., Marnett L. J., Hanawalt P. C. (2004). Malondialdehyde adducts in DNA arrest transcription by T7 RNA polymerase and mammalian RNA polymerase II. *Proceedings of the National Academy of Sciences of the United States of America*.

[137] Eckl P. M. (2003). Genotoxicity of HNE. *Molecular Aspects of Medicine*.

[138] Yang I.-Y., Chan G., Miller H. (2002). Mutagenesis by acrolein-derived propanodeoxyguanosine adducts in human cells. *Biochemistry*.

[139] Hou S.-M., Nori P., Fang J.-L., Vaca C. E. (1995). Methylglyoxal induces hprt mutation and DNA adducts in human T-lymphocytes in vitro. *Environmental and Molecular Mutagenesis*.

[140] VanderVeen L. A., Hashim M. F., Shyr Y., Marnett L. J. (2003). Induction of frameshift and base pair substitution mutations by the major DNA adduct of the endogenous carcinogen malondialdehyde. *Proceedings of the National Academy of Sciences of the United States of America*.

[141] Choudhury S., Pan J., Amin S., Chung F.-L., Roy R. (2004). Repair kinetics of trans-4-Hydroxynonenal-induced cyclic 1,*N*
^2^-propanodeoxyguanine DNA adducts by human cell nuclear extracts. *Biochemistry*.

[142] Huang H., Wang H., Lloyd R. S., Rizzo C. J., Stone M. P. (2009). Conformational interconversion of the *trans*-4-hydroxynonenal-derived (6*S*,8*R*,11*S*) 1,*N*
^2^-deoxyguanosine adduct when mismatched with deoxyadenosine in DNA. *Chemical Research in Toxicology*.

[143] Giunta S., Belotserkovskaya R., Jackson S. P. (2010). DNA damage signaling in response to double-strand breaks during mitosis. *Journal of Cell Biology*.

[144] Burdak-Rothkamm S., Rothkamm K., Prise K. M. (2008). ATM acts downstream of ATR in the DNA damage response signaling of bystander cells. *Cancer Research*.

[145] Zhao H., Traganos F., Darzynkiewicz Z. (2010). Kinetics of the UV-induced DNA damage response in relation to cell cycle phase. Correlation with DNA replication. *Cytometry Part A*.

[146] Cimprich K. A., Cortez D. (2008). ATR: an essential regulator of genome integrity. *Nature Reviews Molecular Cell Biology*.

[147] Agarwal M. L., Taylor W. R., Chernov M. V., Chernova O. B., Stark G. R. (1998). The p53 network. *Journal of Biological Chemistry*.

[148] Kim H. J., Chang S. K. (1998). p53 mutation in patients with ulcerative colitis in rectal biopsy. *The Korean Journal of Internal Medicine*.

[149] Hussain S. P., Amstad P., Raja K. (2000). Increased p53 mutation load in noncancerous colon tissue from ulcerative colitis: a cancer-prone chronic inflammatory disease. *Cancer Research*.

[150] Kotakadi V. S., Jin Y., Hofseth A. B. (2008). Ginkgo biloba extract EGb 761 has anti-inflammatory properties and ameliorates colitis in mice by driving effector T cell apoptosis. *Carcinogenesis*.

[151] El-Abhar H. S., Hammad L. N. A., Gawad H. S. A. (2008). Modulating effect of ginger extract on rats with ulcerative colitis. *Journal of Ethnopharmacology*.

[152] Mirbagheri S. A., Nezami B. G., Assa S., Hajimahmoodi M. (2008). Rectal administration of d-*α* tocopherol for active ulcerative colitis: a preliminary report. *World Journal of Gastroenterology*.

[153] Korenaga D., Takesue F., Kido K. (2002). Impaired antioxidant defense system of colonic tissue and cancer development in dextran sulfate sodium-induced colitis in mice. *Journal of Surgical Research*.

[154] Khanzode S. S., Khanzode S. D., Dakhale G. N. (2003). Serum and plasma concentration of oxidant and antioxidants in patients of *Helicobacter pylori* gastritis and its correlation with gastric cancer. *Cancer Letters*.

[155] Farinati F., Cardin R., Degan P. (1998). Oxidative DNA damage accumulation in gastric carcinogenesis. *Gut*.

[156] Bhagat S. S., Ghone R. A., Suryakar A. N., Hundekar P. S. (2011). Lipid peroxidation and antioxidant vitamin status in colorectal cancer patients. *Indian Journal of Physiology and Pharmacology*.

[157] Yeh C.-C., Lai C.-Y., Hsieh L.-L., Tang R., Wu F.-Y., Sung F.-C. (2010). Protein carbonyl levels, glutathione S-transferase polymorphisms and risk of colorectal cancer. *Carcinogenesis*.

[158] Mehrabi S., Wallace L., Cohen S., Yao X., Aikhionbare F. O. (2015). Differential measurements of oxidatively modified proteins in colorectal adenopolyps. *International Journal of Clinical Medicine*.

[159] Skrzydlewska E., Stankiewicz A., Sulkowska M., Sulkowski S., Kasacka I. (2001). Antioxidant status and lipid peroxidation in colorectal cancer. *Journal of Toxicology and Environmental Health Part A*.

[160] Skrzydlewska E., Sulkowski S., Koda M., Zalewski B., Kanczuga-Koda L., Sulkowska M. (2005). Lipid peroxidation and antioxidant status in colorectal cancer. *World Journal of Gastroenterology*.

[161] Otamiri T., Sjodahl R. (1989). Increased lipid peroxidation in malignant tissues of patients with colorectal cancer. *Cancer*.

[162] Leuratti C., Watson M. A., Deag E. J. (2002). Detection of malondialdehyde DNA adducts in human colorectal mucosa: relationship with diet and the presence of adenomas. *Cancer Epidemiology Biomarkers and Prevention*.

[163] Mistry N., Bevan R. J., Cooke M. S. (2008). Antiserum detection of reactive carbonyl species-modified DNA in human colonocytes. *Free Radical Research*.

[164] Schaeferhenrich A., Beyer-Sehlmeyer G., Festag G. (2003). Human adenoma cells are highly susceptible to the genotoxic action of 4-hydroxy-2-nonenal. *Mutation Research: Fundamental and Molecular Mechanisms of Mutagenesis*.

[165] Pan J., Keffer J., Emami A. (2009). Acrolein-derived DNA adduct formation in human colon cancer cells: its role in apoptosis induction by docosahexaenoic acid. *Chemical Research in Toxicology*.

[166] Eder E., Budiawan (2001). Cancer risk assessment for the environmental mutagen and carcinogen crotonaldehyde on the basis of TD(50) and comparison with 1,N(2)-propanodeoxyguanosine adduct levels. *Cancer Epidemiology Biomarkers and Prevention*.

[167] Eder E., Hoffman C., Bastian H., Deininger C., Scheckenbach S. (1990). Molecular mechanisms of DNA damage initiated by *α*,*β*-unsaturated carbonyl compounds as criteria for genotoxicity and mutagenicity. *Environmental Health Perspectives*.

